# Development of gene transfer for induction of antigen-specific tolerance

**DOI:** 10.1038/mtm.2014.13

**Published:** 2014-04-30

**Authors:** Brandon K Sack, Roland W Herzog, Cox Terhorst, David M Markusic

**Affiliations:** 1 Seattle Biomedical Research Institute, Seattle, Washington, USA; 2 Department of Pediatrics, University of Florida, Gainesville, Florida, USA; 3 Division of Immunology, Beth Israel Deaconess Medical Center, Harvard Medical School, Boston, Massachusetts, USA

## Abstract

Gene replacement therapies, like organ and cell transplantation, are likely to introduce neoantigens that elicit rejection via humoral and/or effector T-cell immune responses. Nonetheless, thanks to an ever-growing body of preclinical studies; it is now well accepted that gene transfer protocols can be specifically designed and optimized for induction of antigen-specific immune tolerance. One approach is to specifically express a gene in a tissue with a tolerogenic microenvironment such as the liver or thymus. Another strategy is to transfer a particular gene into hematopoietic stem cells or immunological precursor cells thus educating the immune system to recognize the therapeutic protein as “self.” In addition, expression of the therapeutic protein in protolerogenic antigen-presenting cells such as immature dendritic cells and B cells has proven to be promising. All three approaches have successfully prevented unwanted immune responses in preclinical studies aimed at the treatment of inherited protein deficiencies, *e.g.*, lysosomal storage disorders and hemophilia, and of type 1 diabetes and multiple sclerosis. In this review, we focus on current gene transfer protocols that induce tolerance, including gene delivery vehicles and target tissues, and discuss successes and obstacles in different disease models.

## The Challenge of Inducing Antigen-Specific Immune Tolerance

**Figure 1:**
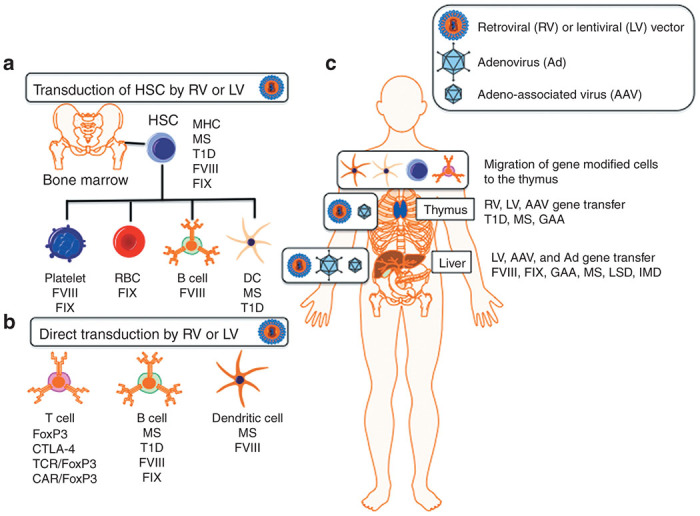
Overview of gene therapy vectors and target cells and tissues for inducing transgene-specific tolerance. (**a**) Hematopoietic stem cells (HSC) transduced *ex vivo* with a retroviral vector (RV) or lentiviral vector (LV) to express an antigen and are transferred to the donor with conditioning to promote engraftment. (**b**) Differentiated T cells, B cells, and dendritic cells (DC) are transduced *ex vivo* with a RV or LV. Antigen-specific expanded effector CD4^+^ T cells are cotransduced with FoxP3 and cytotoxic T-lymphocyte antigen 4 (CTLA-4) to generate Treg. Naive CD4^+^ T cells can be converted into Treg by cotransduction with an antigen-specific T-cell receptor (TCR) or chimeric antigen receptor (CAR) along with FoxP3. B cells are transduced with a RV or LV expressing a transgene-IgG heavy chain fusion protein and transferred back to the donor. DC are cotransduced with a RV or LV expressing the immunosuppressive cytokine IL-10 and a transgene *ex vivo* and transferred back to the donor. (**c**) The thymus and liver are the main target organs for tolerance by *in vivo* gene transfer. Gene-modified HSC and differentiated lineages including DC and T cells are capable of migrating to the thymus and induce nTregs. Direct thymic gene transfer using adeno-associated virus (AAV), RV, or LV results in effector deletion and nTreg induction. Hepatocyte-restricted transgene expression from adenovirus (Ad), AAV, RV, and LV transduction promotes the induction of antigen-specific iTreg. FVIII, factor VIII; FIX, factor IX; GAA, acid-alpha glucosidase; IMD, inherited metabolic disorder; LSD, lysosomal storage disease; MHC, major histocompatibility complex; MS, multiple sclerosis; T1D, type 1 diabetes.

Gene replacement therapy, like organ or cell transplantation, and protein/enzyme replacement therapies share the risk for immune-mediated rejection. The immune system may be induced by the novel antigen(s) to reverse therapy by specific antibody and/or T-cell responses. Another parallel can be drawn with autoimmune diseases, where self-antigens are accidently targeted by antibodies or T cells. Whereas in any of these circumstances the unwanted immune response can potentially be eliminated by general immune suppression, this creates risks for opportunistic infections and typically involves use of drugs with various side effects. Induction of antigen-specific immune tolerance is therefore the preferred choice, which is more likely to succeed when the specifically targeted antigen(s) is known, as general effects on the immune system can be minimized. During the past decade, a number of studies have supported the notion that gene transfer can be a powerful method for inducing antigen-specific tolerance, provided that the requisite-specific vectors, the correct selection of the routes of administration, and target cells are optimized for tolerance induction (Figure 1).

## The Potential for Immune Responses During Gene Therapy

Gene therapy for the correction of monogenic diseases aims at correcting the cause of a disease at the molecular level by delivering a functional copy of a disease-associated defective gene. Viral vectors have emerged as very efficacious delivery vehicles of therapeutic genes to cells *ex vivo* and organs *in vivo*. These vectors have their respective strengths and limitations and are extensively reviewed elsewhere.^1^ One drawback is that the immune system may target antigens associated with the gene transfer vehicle itself. Hence, gene transfer protocols need to be designed to minimize immune responses against the vector itself.

Recently, several new systems have been developed for gene editing, which provides a means for site-specific correction of mutated genes or site-specific insertion of a therapeutic gene with an improved safety profile and the ability to maintain both endogenous tissue and temporal expression. These approaches rely on a synthetic DNA-binding proteins coupled to a dimer-dependent endonuclease (ZFNs and TALENS) or a guide RNA associated with an endonuclease (CRISPR-Cas9) to provide a targeted double-strand break within the mutated gene.^2^ A functional version of the gene, delivered with the endonucleases, can then be edited into the double-strand break using the homologous recombination repair pathway. Next-generation approaches may also combine viral vectors as a delivery platform for site-specific gene editing. Once again, the potential for immune responses against these recombination-mediating proteins or the delivered proteins remain of concern.

In general, immune responses directed against the therapeutic gene product provide a major obstacle to long-term disease correction with gene therapy, especially when the gene product is completely absent. In the case of an absent protein, the newly expressed therapeutic protein is seen by the immune system as nonself, resulting in the activation of both humoral (antibody) and cell-mediated (cytotoxic T lymphocyte) responses. The humoral immune response is often dependent on antigen-specific T-helper lymphocytes (CD4^+^ T cells) to activate B lymphocytes that recognize the same antigen and license their maturation to start producing antibodies. Cell-mediated immunity is directed through the activation of antigen-specific cytotoxic T cells, which are typically major histocompatibility complex (MHC) class I–restricted CD8^+^. Although CD8^+^ T cells can be activated in the absence of CD4^+^ T-helper cells, the speed and strength of activation as well as the generation of a good memory CD8^+^ T response is heavily dependent on help by CD4^+^ T cells. Other subsets of CD4^+^ T cells exist that can dampen or suppress humoral and cell-mediated responses and are dubbed regulatory T cells (Treg). In order to prevent unwanted immune responses against a therapeutic protein, many gene transfer approaches have been developed that selectively activate antigen-specific Treg and induce a state of tolerance.

As explained above, immunological tolerance can be induced at a nonspecific (global immune suppression) or specific antigen-specific level depending on multiple cell intrinsic and extrinsic factors. This review defines tolerance as an active process that maintains unresponsiveness even when repeatedly exposed to antigen (as opposed to immunological ignorance of the antigen). This often involves Treg induction as part of the tolerance mechanism. From a therapeutic standpoint, nonspecific immune suppression is not an optimal approach as it may disrupt normal immune surveillance and responses to antigens, including and not limited to bacterial and viral pathogens and malignant cells. What types of therapies would benefit from inducing antigen-specific tolerance? Gene transfer–based immune tolerance induction protocols can be developed for inherited protein deficiencies, transplant antigens, autoimmune diseases, and allergies.^3^ The focus of this review is to provide an overview on different approaches to promote antigen-specific tolerance through genetic modification or gene transfer to cells and tissues.

## Background: B- and T-Cell Development

Newly generated T lymphocytes undergo maturation and selection within the thymus. In an initial round of selection, immature T cells (CD4^+^ and CD8^+^) are deleted due to a lack of survival signals from the absence of engagement of peptide displayed on the MHC proteins and the T-cell receptor (TCR). Those T cells that receive a threshold level of TCR signaling are further selected for their level of TCR signaling. In this phase, T cells with too strong TCR signaling either undergo receptor editing to modulate affinity or are eliminated through induction of apoptosis. Those T cells that have a moderate level of TCR signaling are retained, complete maturation and released into the circulation. To avoid developing autoimmune responses to tissue-restricted antigens (*i.e.*, antigens not normally expressed in the thymus), medullary thymic epithelial cells express the transcription factor AIRE (autoimmune regulator) that can globally activate mRNA expression. Thus, developing T cells are exposed to most self-proteins, and those with too high of a TCR affinity are eliminated. To underscore the importance of autoimmune regulator in self-tolerance, mice and humans with defective autoimmune regulator suffer from severe autoimmunity.^4^ Similar to T cells, B cells undergo maturation within the bone marrow and later in the spleen and lymph nodes, where highly autoreactive B cells either undergo receptor editing or are deleted.

## Active Immune Suppression by Regulatory T Cells

**Figure 2:**
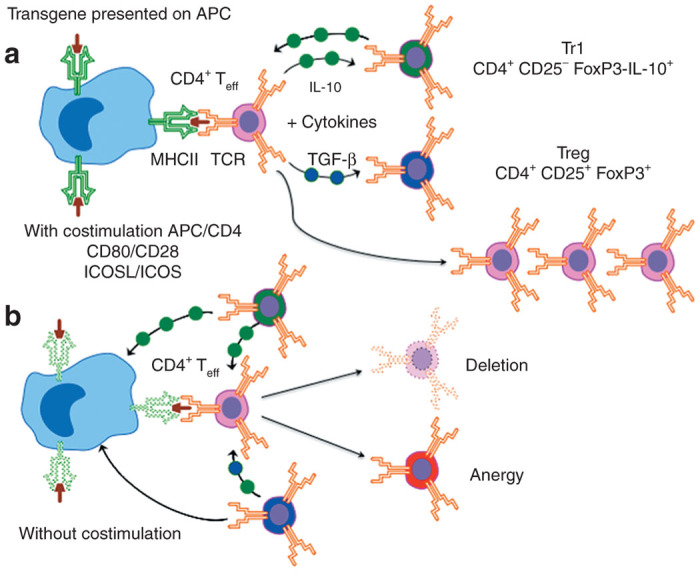
A simplified model for the activation of either antigen-specific Tr1, Treg, or T effector (Teff) cells in the context of TCR engagement of a MHC-II–presented epitope on an APC. (**a**) In the case where there are costimulatory signals between the APC and Teff (CD80/CD28 and ICOSL/ICOS) and the absence of immunosuppressive cytokines, Teff become activated and expand. In the presence of excessive IL-10, Teff can become Tr1 (CD4+CD25−FoxP3−IL-10+) regulatory T cells, and excessive TGF-β promote the induction of iTreg (CD4+CD25+FoxP3+). Tr1 and iTreg can indirectly suppress APC and Teff. In addition, iTreg can also directly suppress APC and Teff through contact inhibition. (**b**) In the absence of costimulatory signals or suppression by Tr1 and Treg, Teff cells are either eliminated or become anergic. TGF, transforming growth factor.

Although thymic selection is effective, some self-reactive T cells escape the selection process. These cells are kept in check via a second level of T-cell immune regulation, which employs a subset of CD4^+^ T cells called regulatory T cells (Tregs). These Tregs, which for this review are minimally defined by the markers CD4^+^CD25^+^FoxP3^+^ (Figure 2), act in an antigen-specific and nonspecific manner to dampen immune responses directly through cell–cell interactions and indirectly by release of immunosuppressive cytokines and sequestration of growth factors. The FoxP3 transcription factor has been identified as a master regulator of Tregs suppressive function and as with autoimmune regulator, severe autoimmunity is associated in mice and humans defective for FoxP3.^5,6^ There are two main subsets of Tregs defined as “central or natural” Tregs (nTregs) developed within the thymus and “peripheral or induced” Tregs (iTregs) developed from peripheral CD4^+^ effector T cells, which are induced to express FoxP3.^7^ Although studies indicate there are little differences in suppressive function between nTreg and iTreg, there are some indications that nTreg exhibit more stable FoxP3 expression, whereas iTreg are considered to be more plastic and can lose FoxP3 expression and revert back to effectors.^8^ Phenotypic differentiation of nTreg and iTreg can be difficult. Some reports indicate that Helios or Neuropilin-1 are exclusively expressed in nTregs and that nTregs are more hypomethylated in the Treg-specific demethylated region. Although there are other classes of suppressive T cells such as T regulatory 1 (Tr1) cells defined as IL-10 secreting CD4^+^ (Figure 2) and CD8^+^ T cells,^9,10^ CD8^+^ FoxP3^+^ T cells,^11^ and IL-10^+^ regulatory B cells (Breg),^12–15^ such cells and their role in antigen-specific tolerance through gene transfer are beyond the scope of this review.

## Tolerance Induction by Hepatocyte-Restricted Transgene Expression

### Liver tolerance and the hepatic environment

One pathway to specific tolerance induction is to express the antigen in a tissue that is prone to activation of immune regulatory pathways. The potential of using the liver as an organ for promoting tolerance was initiated from early transplant studies conducted in MHC-mismatched animals and humans.^16–18^ Further evidence came from basic anatomy and physiology studies that places the liver immediately downstream of blood flow from the gut, where the liver is routinely exposed to copious amounts of foreign antigens derived from food and bacteria. The fact that liver transplants are well tolerated compared with other single organ transplants, that multiple organ transplants from the same donor are better tolerated when the liver is transplanted, and that eating a meal does not routinely induce severe inflammation in the liver suggested that there is a mechanism in place for dampening immune responses. Indeed, many pathogenic viruses and parasites are able to exploit this mechanism and develop protection from immune-mediated clearance as seen with chronic infections of hepatitis B and C viruses and malaria, which initially infects human hepatocytes.^19^

The liver primarily consists of the following cell types: hepatocytes, resident macrophages (Kupffer cells), specialized endothelial cells, liver sinusoidal endothelial cells, and hepatic stellate cells. Each of these liver cells has been associated with contributing to tolerance independently and most likely act synergistically to skew local immune responses toward tolerance.^20–32^ Thus, it is possible to induce transgene-specific tolerance by liver gene transfer and expressing the transgene in this microenvironment. When designing a tolerogenic, liver-directed gene transfer protocol, several critical factors have to be considered, including: immunological microenvironment of the liver, restricted transgene expression to hepatocytes, achieving adequate levels of transgene expression, the relative immunogenicity of the gene transfer vehicle and transgene, and optimal induction of Treg.

### Tolerance induction to transgene products by *in vivo* viral vector gene transfer to hepatocytes

Published protocols that were successful in tolerance induction typically used *in vivo* gene transfer mediated by a viral vector, with expression restricted to hepatocytes. Among these, adeno-associated viral (AAV) vectors have been used to induce tolerance to a large number of transgene products. AAV vectors have the advantages of the availability of serotypes with strong tropism to hepatocytes and of limited innate immunogenicity.^33,34^ When initial inflammatory responses are low, activation signals to the immune system may be avoided, thereby increasing the chance for transgene expression to induce tolerance. Similarly, limited induction of IFN I (IFNα/β) preserves transgene expression and reduces antiviral responses.^35^ A minimal level of transgene expression is required for tolerance induction, for example, ~1% of normal coagulation factor IX levels in murine models.^32^ For therapeutic transgenes that have low levels of expression, such as factor VIII protein, it may be possible to employ codon optimization of the cDNA encoding the transgene product to augment protein expression to a level that promotes tolerance.^36,37^ One of the key mechanistic features of tolerance induction by hepatic gene transfer is the induction of transgene product-specific CD4^+^CD25^+^FoxP3^+^ Treg.^38–40^ Induced Treg actively suppress antibody and CD8^+^ T-cell responses against the transgene product.^31,38,41^ Treg induction is required for induction and maintenance of tolerance and correlated to the level of transgene expression.^38,39,42^ Although secreted antigens may also be presented in the thymus, peripheral Treg induction, a transforming growth factor-β–dependent process, is likely a major source for the generation of transgene product-specific Treg.^21^ The costimulatory molecule glucocorticoid-induced tumor necrosis factor receptor ligand has recently been identified as another factor required for efficient induction of Treg following AAV liver gene transfer.^43^ For suppression of CD8^+^ T-cell responses, hepatic expression of the suppressive cytokine IL-10 is required, which occurs in Kupffer cells and in Treg such as the aforementioned FoxP3^+^ Treg or type 1 regulatory (Tr1) T cells, which are IL-10–induced CD4^+^CD25^−^FoxP3^−^ cells expressing transforming growth factor-β and large amounts of their hallmark cytokine IL-10.^21,27^ Activation of CD8^+^ T cells may be further reduced by the development of vector genomes devoid of immune stimulatory CpG motifs, as innate immunity to AAV vectors in the liver is TLR9 dependent.^44,45^ In addition to Treg induction, deletion of effector T cells, via induction of activation-induced cell death/programmed cell death, has been shown to be required for effective tolerance induction.^32,46,47^

The importance of hepatocyte-restricted expression for tolerance is underscored in a set of studies evaluating gene transfer with a lentiviral vector (LV), an alternative platform for hepatic tolerance induction. LV pseudotyped with the VSVg envelope protein efficiently transduce antigen-presenting cells (APC) in the liver^48^ and fail to induce tolerance even when using tissue-specific promoters. Remarkably, when transgene expression is detargeted in APC by a complementary microRNA target to microRNA 142-3p, a microRNA specifically expressed in hematopoietic cells, tolerance is induced for cytosolic GFP^49^ and secreted hFIX protein for conventional LV^50^ and integrase-defective LV.^51^ Hence, although we understand some of the factors needed for tolerance, certain questions such as, what are the tolerogenic APCs in the liver, remain unanswered. Additional details on the mechanism for tolerance by liver gene transfer have been extensively reviewed elsewhere.^16,52,53^

Illustrating the broad applicability of the approach, liver gene transfer has resulted in the induction of robust transgene tolerance to a variety of cytosolic and secreted transgene products in small and large animal disease models, including hemophilia A,^36,37,54–57^ hemophilia B,^32,39,42,58,59^ Pompe disease,^60–62^ allo-MHC for skin graft,^63^ and experimental autoimmune encephalomyelitis.^64^ Liver-induced tolerance extends to extrahepatic tissues, such as muscle, brain, and central nervous system as demonstrated in supplemental gene transfer to the muscle,^65^ and to brain/central nervous system in Niemann–Pick disease,^66^ central nervous system in experimental autoimmune encephalomyelitis,^64^ and muscle in Pompe disease.^62^ Hence, in an optimized protocol, immune tolerance induced by hepatic gene transfer may be dominant over activation of immune responses elsewhere, a phenomenon that can be exploited for treatment of disease that requires gene transfer to multiple organs or for development of immune modulatory gene therapy. Such a protocol may involve coadministration of a liver-targeted vector with a second vector targeting other tissues or simultaneous or sequential administration of two vectors via different routes. There are now a growing number of published studies demonstrating long-term correction of a variety of inherited metabolic and lysosomal storage disorders following liver gene transfer^67,68^ and supporting evidence in nonhuman primates that AAV8 liver-directed α-galactosidase A promotes tolerance.^69^

Although there have been ample studies in different disease models showing that liver gene transfer can prophylactically induce transgene tolerance, there have been limited studies on the potential for hepatic gene transfer to reverse an ongoing immune response. In the case of hemophilia A and B, patients with severe forms of disease are at risk to develop inhibitory antibodies against FVIII and FIX proteins during the course of recombinant protein therapy. It is unknown what the impact on patients with inhibitors would be following liver gene transfer. In the case of hemophilia B and/or Pompe disease, a subset of patients who develop inhibitory antibodies during enzyme replacement therapy to FIX and GAA proteins develop acute anaphylaxis. One could predict that liver gene transfer would either exacerbate or suppress the ongoing immune response. Three recent studies addressed this important question in a canine hemophilia A^70^ and murine hemophilia B models.^42,71^ Importantly, each of these studies indicated that liver gene transfer with an AAV or LV could reverse preexisting inhibitors, provide therapeutic factor expression, and protect against anaphylaxis and pathogenic antibody responses. Inhibitor reversal was dependent on the active suppression of induced antigen-specific Treg that rapidly eliminated antibody-secreting plasma cells and suppressed the activation of memory B cells. Thus, beyond therapeutic protein expression, liver-directed gene transfer might hold promise as a novel approach to treating autoimmune disease and severe allergies.

AAV vectors, which are largely maintained in episomal form, have now been successfully used in clinical trials for liver gene transfer. Due to size limitations, it is difficult to include extensive endogenous enhancer and promoter elements within the vector to maintain regulated, spatial, and temporal therapeutic transgene expression. Although strict control of transgene expression is not as critical for secreted zymogens, such as FVIII and FIX in hemophilia, other therapeutic transgenes may require strict regulation. Taking advantage of new gene-editing tools, Li *et al*.^72^ demonstrated robust gene correction in the liver of young hemophilia mice using AAV vectors to deliver a specific ZFN and *hF9* cDNA sequence. This study was followed by similar results by Anguela *et al*.^73^ in adult hemophilia mice. Such an approach is also able to direct site-specific integration into so-called “safe harbor” regions within a chromosome and provided stable transgene expression with minimal genotoxicity, such as a reduced risk for insertional mutagenesis, and may pave the way as a next-generation therapeutic for treating monogenic disorders.

## Taking Advantage of Age-Dependent Development of the Immune System—Neonatal and *IN UTERO* Gene Transfer

Another approach that has seen some success in tolerance induction is liver gene transfer either *in utero* or neonatally. The idea is that expressing a transgene when the immune system is immature or in the early stages of development will promote tolerance, most likely in a mechanism that incorporates the transgene as a self-protein. Additionally, this approach would also avoid any potential immune responses directed against the delivery vector. Naturally, such an approach is more effective with a gene delivery system that provides stable integration of the transgene cassette (such as retrovirus, lentivirus, or site-directed integration) as episomal vectors will become diluted and eventually lost as the liver grows to adult size. Most successes with neonatal gene transfer tolerance are in murine models, as mice have a very immature immune system at birth and in some reported canine studies.^55,74–78^ Although there have been many neonatal gene transfer studies conducted in rats and large animal models (cats, dogs, and nonhuman primates), tolerance induction is often not as robust as seen in mice.^79–82^

## Thymic Gene Transfer—Negative Selection and NTREG Induction

Given the role of the thymus in the negative selection of autoreactive T cells and induction of nTreg, it is not surprising that direct thymic gene transfer has been considered as a means of inducing antigen-specific tolerance.^83^ Such studies conducted in mice demonstrated induction of specific tolerance to viral antigens,^84–86^ reduction in the occurrence of type 1 diabetes in nonobese diabetes mice,^85^ protection against the development but not progression of experimental autoimmune encephalomyelitis,^87^ and resistance to the therapeutic hGAA protein.^88^ Alternatively, Hadeiba *et al*.^89^ have demonstrated that CCR9-expressing plasmacytoid dendritic cells (DCs) can be peripherally loaded with an antigen and migrate to the thymus to promote tolerance. Although such an approach may be feasible in animal models, the exact mechanism that determines whether a T cell becomes an effector or regulatory T cell following encounter with MHC-II–presented antigen is not completely understood.^90–92^ Therefore, some “fine tuning” may be required in designing a gene transfer approach that can reliably promote induction of antigen-specific nTreg.

## Hematopoietic Stem Cell Gene Transfer for Transplant Tolerance, Treatment of Inherited Protein Deficiencies, and Autoimmune Disease

Hematopoietic stem cells (HSCs) represent an attractive target cell for genetic modification for tolerance induction. Defined protocols have been established for the collection, culturing, transduction, and transfer/engraftment into a recipient. In most instances, autologous cells can be used, reducing potential host versus graft disease. Using specific regulatory elements such as tissue-specific promoters and microRNA targets, it is possible to strictly control transgene to a particular cell lineage.^93–97^ As platelets, lymphocytes (B and T cells), and most of our professional APC are derived from HSC, it is possible to direct the expression of a transgene product to promote the generation of nTreg from antigen presentation in the thymus or peripheral induction of CD4^+^ effector T cells to iTreg. Later sections will discuss approaches of direct gene modification of differentiated B and T cells and professional APCs. Therefore, it is not surprising that similar to the liver, hematopoitic stem cell gene transfer has been used for expressing therapeutic proteins and for inducing transgene-specific tolerance.

HSC gene modification for inducing tolerance was inspired by the observation of immunological tolerance to donor MHC proteins following the generation of mixed donor–host chimerism following HSC transplantation.^98,99^ Although this approach could induce tolerance to donor cells and tissues,^100^ use of allogenic HSC often resulted in graft versus host disease and engraftment failure. Therefore, to prevent graft versus host disease, investigators found a way to generate molecular chimerism by autologous HSC gene transfer.^101–106^ Gene transfer to HSC has successfully induced tolerance for tissue transplantation, desensitized allergic responses, protected against autoimmune diseases, and provided tolerance and therapeutic protein expression in a variety of disease models.^102,107–110^

One of the limiting factors for successful tolerance induction of gene-modified HSCs is efficient engraftment into the host. Efficient engraftment in early HSC transplantations often required complete myeloablation of the host bone marrow compartment by total body irradiation. Milder nonmyeloablation conditioning regimens using chemicals or low-dose radiation often failed to promote sufficient levels of engraftment to induce tolerance but instead were hyporesponsive,^102,107^ with the level of antigen expression determining hyporesponsiveness or tolerance. Newly developed nonmyeloablative regimens and gene transfer platforms can now provide sufficient engraftment and transgene expression for successful tolerance induction from gene-modified HSC following transplantation.^111–114^

Genetic modification of HSCs has been used for the induction of tolerance toward skin grafts using the cytosolic reporter gene GFP^115,116^ and MHC-II.^117^ In terms of controlling autoimmunity, gene transfer to HSC has been successful in preventing onset and controlling early disease progression in an experimental autoimmune encephalomyelitis mouse model for multiple sclerosis^118^ and recently has been shown to be effective using nonmyeloablative conditioning to effectively halt disease progression.^112^ Additional success has been obtained in controlling progression of type 1 diabetes in a nonobese diabetes mouse model.^109^ HSC gene modification has also been reported to control allergic responses in a mouse model.^95,111,119^ Several small and large animal disease models have shown long-term correction and tolerance using HSC gene transfer protocols including hemophilia A,^120–125^ hemophilia B,^126–129^ and Pompe disease.^130,131^ The recently reported safety and efficacy of LV gene transfer to HSCs in two clinical trials for Wiskott–Aldrich syndrome^132^ and metachromatic leukodystrophy^133^ provide optimism for the translation of some of the above studies into new clinical trials.

## B-Cell Gene Transfer for Tolerance Induction

In addition to producing antibodies, B cells are also APCs, particularly for memory CD4^+^ T cells. Interestingly, it is possible to harness the ability of B cells to process and present antigen, not only to promote immune responses but also for tolerogenic antigen presentation. Specifically, stable retroviral gene transfer to primary B cells of a transgene fused in frame to the immunoglobulin G (IgG)-1 heavy chain leads to the induction of antigen-specific iTreg and tolerance.^134–136^ In this method, tolerance induction was shown to be dependent on endogenous processing and MHC-II presentation of the fusion gene product,^135^ B7 expression on B cells,^137^ and on CD4^+^CD25^+^FoxP3^+^ Treg.^138,139^ Expression of the immunosuppressive cytokine IL-10 may be required in gene-modified B cells (possibly through induction of regulatory Tr1 cells), as suggested by one study or indirectly required in cells of the recipient of the B-cell therapy as suggested by others.^140–142^ It is interesting to note that multiple MHC-II epitopes dubbed “Tregitopes” have been identified within the Fc fragment of IgG that expand nTreg and promote global tolerance.^143^ Tregitopes have been used to promote antigen-specific tolerance,^144–148^ likely through a bystander suppression mechanism.

Gene-modified B cells expressing antigens fused to IgG has provided antigen-specific tolerance in autoimmune models^149^ including multiple sclerosis,^150,151^ rheumatoid arthritis,^152^ and type 1 diabetes.^150^ In addition to autoimmune disease, retroviral gene transfer of an in-frame fusion of FVIII or FIX to IgG1 heavy chain to B cells is capable of promoting tolerance and controlling inhibitors in murine hemophilia A and B.^153,154^ As seen with liver gene transfer of FIX protein, FIX-IgG–expressing B cells are capable of partially reversing ongoing anti-FIX immune responses and can protect against anaphylaxis.

## T-Cell Gene Modification and Tolerance

Following the identification of FoxP3 as a master regulator for Treg,^5,155,156^ it was demonstrated that forced expression of FoxP3 by retroviral gene transfer to effector CD4^+^ T cell produced cells with similar suppressive functions to Treg.^157^ A typical protocol consists of expanding CD4^+^ effector T cells *ex vivo* followed by transduction with a retroviral vector expressing FoxP3 and has been successful in inducing tolerance in graft versus host disease^158^ and autoimmune disease.^159,160^ Monoclonal expanded CD4^+^ effector T cells are more efficient than polyclonal cells following FoxP3 transduction for inducing antigen-specific tolerance. Additional forced expression of other Treg surface markers such as CTLA-4 can further improve suppressive function. Additionally, it is possible to generate antigen-specific Treg by gene transfer of an antigen-specific TCR^161^ to nTreg or combining gene transfer of an antigen-specific TCR and FoxP3 to naive CD4^+^ T cells.^162^ Chimeric antigen receptors have also been tested recently as a means for generating antigen-specific Treg and provided protection in a murine multiple sclerosis model.^163^ It is not clear if signaling through the chimeric antigen receptors is activating Tregs upon encountering antigen or providing a means of enriching the local concentration of Treg. Future studies are required to define the mechanism of tolerance with chimeric antigen receptor–modified Treg.

## DC Gene Modification

DCs as professional APCs are capable of presenting antigen on MHC-II and depending on their maturation state can activate either CD4^+^ effector T cells or Tregs.^164^ Therefore, gene transfer of an antigen to immature DCs is a potential approach to inducing antigen-specific Treg. Given the fact that DCs contain sensors for viral pathogen–associated molecular patterns, finding a means to transduce DC with a viral vector without inducing maturation has proven challenging.^165,166^ Nonetheless, a successful protocol was reported generating FVIII-specific Treg by *ex vivo* transduction of tolerogenic DC that express either FVIII or FVIII and IL-10.^167^ A second approach used *in vivo* gene transfer of LV with a DC-restricted promoter to promote central and peripheral antigen-specific tolerance.^168^ Although direct modification of DCs is successful for inducing antigen-specific tolerance, gene transfer to a precursor such as HSCs, coupled with antigen-restricted expression to a DC lineage, may prove more effective.^169^

## Conclusions

The induction of transgene-specific tolerance through gene transfer or gene modification is possible using a variety of cells and tissues and gene delivery vehicles. Tolerance is typically dependent on the induction of antigen-specific Treg. Preclinical studies conducted in rodent and canine disease models have demonstrated robust tolerance induction, the ability to transfer tolerance by adoptive transfer of Treg to naive animals, and the ability to suppress ongoing immune responses. The fact that liver gene transfer can induce Treg in a “hostile” proinflammatory setting and mediate suppression in the midst of an ongoing immune response has broader implications beyond therapies aimed at treating monogenic disorders and offer therapeutic approaches to treat autoimmune and hypersensitivity disorders. Clinical studies evaluating *ex vivo* gene delivery to HSC and *in vivo* gene transfer to muscle and liver have so far demonstrated no immune responses directed against the therapeutic gene product, suggesting that humans may respond similarly as seen in preclinical studies. Indeed, most immunological complications in patients have been associated with immune responses directed against the gene delivery vector and vector-associated genotoxicity. Although beyond the scope of this review, it is also important to note that including a transient immune suppression protocol that spares Treg can augment tolerance mediated by gene transfer, especially when using highly immunogenic vectors, delivery routes, or transgenes,^170–172^ Conversely, careful consideration should be placed on avoiding immune suppression protocols that effect Treg, as this can induce unwanted transgene immune responses.^173^ The advent of new tools for site-specific modification of chromosomes may greatly reduce the risks for insertional mutagenesis and pave the way for a transition from gene therapy to one of gene editing.
